# High risk of macrosomia in newborns of immigrant mothers

**DOI:** 10.1186/s13052-020-0771-2

**Published:** 2020-01-16

**Authors:** Mario De Curtis, Leonardo Villani, Arianna Polo

**Affiliations:** 1 Maternal and Child Health Department, University of Rome La Sapienza, Piazzale Aldo Moro, 5, 00185 Rome, Italy; 2 Public Health Department, Università Cattolica del Sacro Cuore, Rome, Italy; 3 Direzione Salute e Integrazione Sociosanitaria Regione Lazio, Rome, Italy

**Keywords:** Macrosomia, Immigrant, Inequality

## Abstract

**Background:**

In Italy live about 8.7% immigrants, which contribute to more than 15% of all deliveries taking place in Italy. We aimed to investigate whether newborns from high migratory pressure countries (HMPC) mothers have a different macrosomia and post-term pregnancy incidence compared to Italian newborns.

**Methods:**

In this retrospective observational study, we analyzed data on 404.863 babies born between 2010 and 2017. Italian mothers delivered 309.658 (76.5%), HMPC mothers 88.179 (21.8%) and developed country (DC) mothers 7.026 (1.7%) babies. We analyzed the incidence of macrosomia and post term pregnancy.

We estimated incidence rate (IR), unadjusted incidence rate ratio (IRR) and 95% confidence intervals (CIs) to evaluate the association between these perinatal parameters and the mother’s region of birth.

**Results:**

HMPC compared to Italian newborns showed a significantly higher incidence of birthweight > 4000 g (53.3‰ vs 39.1‰, *p*-value < 0.001; IRR 1.4, 95%CI = 1.36–1.45), birthweight ≥4500 g. (7.0‰ vs 3.8‰, p-value < 0.001; IRR 1.8, 95%CI = 1.67–2.0) and gestational age at birth > 41 weeks (19.9‰ vs 12.8‰, p-value < 0.001; IRR 1.55, 95%CI = 1.47–1.64).

The macrosomia incidence between HPMC and Italian newborns was significantly increased at all gestational ages (Fig. 1), especially for mothers coming from Central Eastern Europe (121.79‰ vs 91.1‰, *p*-value< 0.001; IRR 1.34, 95%CI = 1.11–1.62).

**Conclusion:**

In Italy immigrant status represents a risk factor for macrosomia and post-term birth, which could be related to socio-economic status and unfavorable life conditions of immigrant mothers during pregnancy.

## Main text

In Italy live about 8.7% immigrants, which contribute to more than 15% of all deliveries taking place in Italy. This estimate substantially increases considering newborns from foreign mothers with Italian fathers [1]. Immigrant mothers encounter during gestation and delivery several problems and newborns are very often premature [2]. We aimed to investigate whether newborns from high migratory pressure countries (HMPC) mothers have a different macrosomia and post-term pregnancy incidence compared to Italian newborns.

In this retrospective observational study, we obtained data from the Lazio hospital discharge database, which records perinatal information on all newborns. The Lazio Region registers each year 10% of all newborns delivered in Italy. We analyzed data on 404.863 babies born between 2010 and 2017. Italian mothers delivered 309.658 (76.5%), HMPC mothers 88.179 (21.8%) and developed country (DC) mothers 7.026 (1.7%) babies. We identified eight regions of origin within the HMPC group. We analyzed the incidence of macrosomia (birth weight > 4000 g. or ≥ 4500 g.) and post term pregnancy (> 41 weeks gestational age).

We estimated incidence rate (IR), unadjusted incidence rate ratio (IRR) and 95% confidence intervals (CIs) to evaluate the association between these perinatal parameters and the mother’s region of birth. The comparison was carried out between HMPC and Italian newborns because we considered DC newborns similar to Italian babies.

HMPC compared to Italian newborns showed a significantly higher incidence of birthweight > 4000 g (53.3‰ vs 39.1‰, *p*-value < 0.001; IRR 1.4, 95%CI = 1.36–1.45), birthweight ≥4500 g. (7.0‰ vs 3.8‰, p-value < 0.001; IRR 1.8, 95%CI = 1.67–2.0) and gestational age at birth > 41 weeks (19.9‰ vs 12.8‰, *p*-value < 0.001; IRR 1.55, 95%CI = 1.47–1.64).

**Fig. 1:**
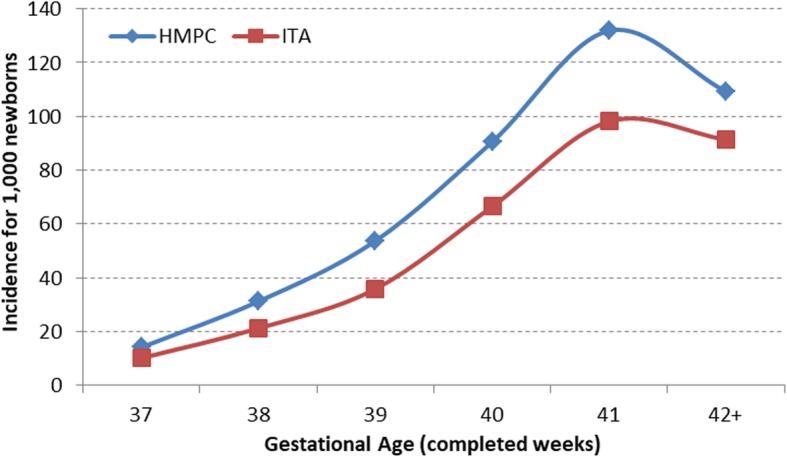
Incidence of macrosomia (birthweight>4000 gr) in newborns of mothers coming from high migratory pressure countries (HMPC) and infants born to Italian mothers (ITA)

The macrosomia incidence between HPMC and Italian newborns was significantly increased at all gestational ages (Fig. 1), especially for mothers coming from Central Eastern Europe (121.79‰ vs 91.1‰, p-value< 0.001; IRR 1.34, 95%CI = 1.11–1.62).

Higher obesity incidence in foreign mothers’ and gestational diabetes, favorited by a high glycemic diet, possibly due to lower costs of these foods, might explain those differences [3].

In Italy immigrant status represents a risk factor for macrosomia and post-term birth, which could be related to socio-economic status and unfavorable life conditions of immigrant mothers during pregnancy.
